# Pharmacologic properties and inhibitory activity of 6-azasteroids against *Mycobacterium leprae in vivo* and *in vitro*

**DOI:** 10.1128/spectrum.00228-25

**Published:** 2025-05-27

**Authors:** Thabatta L. S. A. Rosa, Maria Angela M. Marques, Tianao Yuan, Daniele F. F. Bertoluci, Joshua Werman, Fabricio da M. R. Costa, Linda A. Fischbacher, Mariana de Andrea Hacker, Ramanuj Lahiri, Márcia Bêrredo-Pinho, Patrícia S. Rosa, John T. Belisle, Nicole S. Sampson, Maria Cristina V. Pessolani

**Affiliations:** 1 Laboratório de Microbiologia Celular, Instituto Oswaldo Cruz, Fundação Oswaldo Cruz 37903, Rio de Janeiro, State of Rio de Janeiro, Brazil; 2 Department of Microbiology, Immunology and Pathology, Colorado State University 164597 , Fort Collins, Colorado, USA; 3 Department of Chemistry, Stony Brook University 603074, Stony Brook, New York, USA; 4 Divisão de Pesquisa e Ensino, Instituto Lauro de Souza Lima 368269, Bauru, State of São Paulo, Brazil; 5 Laboratório de Hanseníase, Instituto Oswaldo Cruz, Fundação Oswaldo Cruz 37903, Rio de Janeiro, State of Rio de Janeiro, Brazil; 6 National Hansen’s Disease Programs, Laboratory Research Branch, School of Veterinary Medicine, Louisiana State University 70164, Baton Rouge, Louisiana, USA; 7 Department of Chemistry, University of Rochester 429052, Rochester, New York, USA; Central Texas Veterans Health Care System, Temple, Texas, USA

**Keywords:** *Mycobacterium leprae*, cholesterol, cholestenone, 3β-HSD, 6-azasteroids, chemotherapy, pharmacokinetics

## Abstract

Although the current multidrug therapy (MDT) for leprosy is very successful, the long treatment duration and the emergence of antibiotic-resistant strains demand for new alternative drugs. One potential target for drug development against pathogenic mycobacteria is their need to degrade host cholesterol during infection. *Mycobacterium leprae*, due to its degenerate genome, has preserved only the first step of cholesterol catabolism, in which cholesterol is oxidized to cholestenone by the enzyme 3β-hydroxysteroid dehydrogenase (3β-HSD). *M. leprae* avidly produces cholestenone *in vivo,* and this metabolic activity seems to play an important role in bacterial pathogenesis. In this study, six 6-azasteroid analogs were developed with the potential to inhibit 3β-HSD, and their metabolic stability and *in vitro* and *in vivo* inhibitory activity against *M. leprae* were investigated. Pharmacologic properties indicated lower metabolic liabilities for azasteroid **5** in comparison to its progenitors, azasteroids **1** and **2,** resulting in improved accumulation and extended *t*_1/2_ values in mice. Azasteroid **5** also partially inhibits *M. leprae* 3β-HSD *in vitro*. When tested in the Shepard’s mouse footpad model of leprosy, azasteroid **5** showed effective bacterial killing that was accelerated when combined with a subinhibitory dose of rifampicin and exhibited an absence of detectable hepatotoxic effects. We concluded that 6-azasteroids derivatives are promising new antimicrobial candidates for leprosy treatment.

**IMPORTANCE:**

Leprosy remains a significant global health challenge, particularly in underserved regions. While multidrug therapy (MDT) has been effective, its prolonged duration and the emergence of antibiotic-resistant strains emphasize the urgent need for novel therapeutic strategies. Recent advances in understanding *Mycobacterium leprae*’s unique biology have identified cholesterol metabolism as a critical pathway for bacterial survival and pathogenesis, offering a promising new target for drug development. Building on insights from tuberculosis research, azasteroids—compounds known for their potential to disrupt mycobacterial cholesterol metabolism—are now being explored as candidates for leprosy treatment. These molecules inhibit *M. leprae*’s cholesterol oxidation, impairing bacterial persistence within the host. This innovative approach could lead to more effective, faster-acting therapies, overcoming current treatment limitations and resistance. Such efforts represent a vital step forward in reducing the burden of leprosy and empowering affected communities worldwide.

## INTRODUCTION

Leprosy is a tropical neglected disease caused mainly by the obligate intracellular bacteria *Mycobacterium leprae*. The disease remains a public health problem in several countries, including Brazil and India (1). Multi-drug therapy (MDT), which consists of a combination of rifampicin, dapsone, and clofazimine, has been effective in combating the infection drastically decreasing leprosy global prevalence in the past four decades (1). Despite the success of MDT, the strategy is hindered by long treatment duration, the emergence of antibiotic-resistant strains (2), and the report of several adverse effects in patients under MDT (3). Thus, there is a demand for alternative antimycobacterial drugs for leprosy to achieve disease elimination.

A promising therapeutic target in mycobacteria is the cholesterol catabolism pathway (4, 5). Mycobacteria, in general, including *Mycobacterium tuberculosis*, catabolize cholesterol and use it as a source of carbon and energy (6–8). However, due to its degenerate genome, *M. leprae* has preserved only the first step of cholesterol catabolism, avidly incorporating cholesterol and oxidizing it to cholestenone (9). In a recent report, we showed that *M. leprae* 3β-hydroxysteroid dehydrogenase (3β-HSD), coded by the gene *ml1942*, is the enzyme responsible to this reaction (10). Importantly, we showed that 3β-HSD activity generates reductive energy such as NADH and NADPH that can be used by *M. leprae* for ATP synthesis and the biosynthesis of key lipid virulence factors. Thus, the 3β-HSD of *M. leprae* provides an important metabolic function and participates in bacterial pathogenesis (10).

The 3β-HSD of *M. tuberculosis* also uses cholesterol as substrate and NAD^+^ as the main enzyme cofactor and is inhibited by trilostane (11)*,* a steroid analog that is often used as an inhibitor of the human 3β-HSD (12). Thus, we sought to develop compounds with the potential to inhibit the utilization of cholesterol by *M leprae*. For this purpose, we chose to focus on azasteroids, specifically 4- and 6-azasteroids, which are cholesterol analogs with nitrogen at the 4- or 6-position in the steroidal nucleus. 6-Azasteroids were originally developed by Glaxo Wellcome for two non-anti-infectious-disease indications (benign prostatic hyperplasia and male pattern baldness) (13, 14). 6-Azasteroids potently inhibit mammalian type 1 and type 2 5α-reductase that catalyze the conversion of testosterone to a more potent androgen, dihydrotestosterone. 6-Azasteroids displayed excellent pharmacokinetic/pharmacodynamic properties and a wide therapeutic safety index in rats and dogs (15). We hypothesized that compounds based on the repurposed 6-azasteroid-scaffold would have potential as inhibitors of cholesterol metabolism in mycobacteria. During the course of our studies on synergistic inhibitors in *M. tuberculosis*, we identified 12 azasteroids that inhibit 3β-HSD activity in *M. tuberculosis* and *M. leprae* (10, 16, 17). In a recent report, we tested one of these 6-azasteroids (azasteroid **1**) *in vitro* against *M. leprae* and showed inhibition of cholestenone production and the killing of intracellular bacilli (10). In the present study, additional 6-azasteroid derivatives were synthesized, and their metabolic stability and *in vitro* and *in vivo* inhibitory activity against *M. leprae* were investigated.

## MATERIALS AND METHODS

### 6-azasteroid synthesis

**Fig 1:**
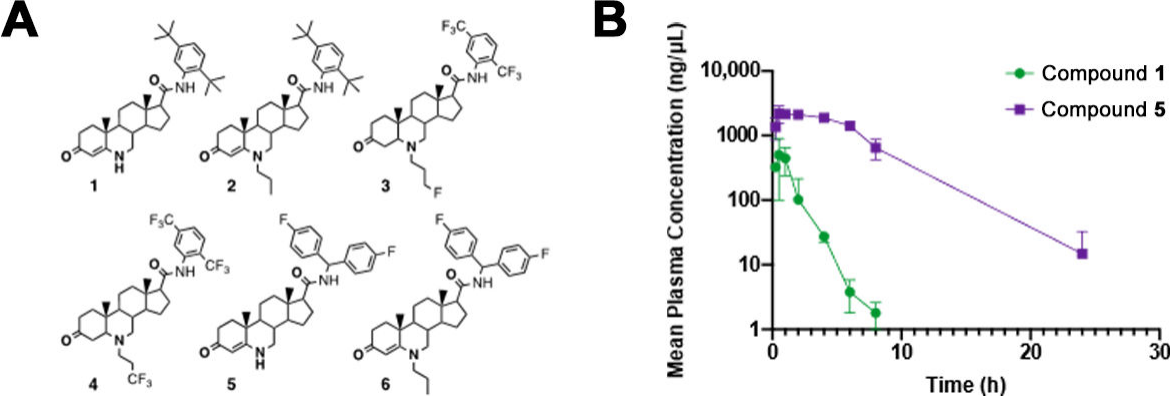
(**A**) Structures of 6-azasteroids used in this work. (**B**) Assessment of *in vivo* half-lives of compounds 1 and 5. CD1 male mice (8 weeks, 20–30 g) were treated with 5 mg/kg via oral gavage and sacrificed at designated time points. Plasma was extracted, and the concentration of each 6-azasteroid was determined by liquid chromatography–tandem mass spectrometry. Values are averages of three technical replicates, and error bars indicate the standard deviation.

6-Azasteroids **1–6** (Fig. 1A) were synthesized and purified as previously described (13, 14, 17, 18). All the products were purified to >95% purity by column chromatography or high-performance liquid chromatography.

### Determination of IC_50_ values for 6-azasteroids’ cytotoxicity

THP-1 cells and HepG2 cells were utilized to assess the cytotoxicity of 6-azasteroids by means of the method outlined by Miret et al. (19). The cells were purchased from ATCC, USA (HB-8065 and TIB-202), and were cultured from frozen stock grown on Eagle’s Minimum Essential Medium (Corning, USA) or Roswell Park Memorial Institute 1640 medium (Corning) supplemented with 10% fetal bovine serum (FBS) and 0.1% penicillin/streptomycin. Cells were plated in 96-well plates at 10^4^ cells/well. The azasteroids were dissolved in DMSO to yield a 20 mM stock solution, and serial dilutions were prepared. Assays were performed in technical triplicates. The azasteroids were incubated with cells for 72 h, after which Alamar Blue staining was performed. Data were analyzed by determining the concentration of oxidized Alamar Blue in each well. Results were plotted in a dose–response curve against the log of 6-azasteroid concentration and fitted to a nonlinear curve. IC_50_ values are reported as micromolar drug concentrations.

### Determination of *in vitro t*_1/2_ values of 6-azasteroids in human liver microsomes

Experiments were performed by Pharmaron, Inc., China. To determine the microsomal intrinsic clearance, each 6-azasteroid (1 µM) was incubated with human liver microsomes (0.5 mg/mL, taken from a mixed-gender pool) at 37 °C for 1 h in 100 mM phosphate buffer (pH 7.4) containing 5 mM MgCl_2_ and 1 mM NADPH. Negative control samples were prepared by replacing the NADPH solution with ultrapure H_2_O. The negative controls were used to exclude the confounding variable of test compound instability. Experiments involving NADPH were carried out in duplicate. Negative controls were prepared in singlet. Aliquots (90 µL) were taken at 0, 15, 30, 45, and 60 min of incubation. The microsomal reaction in each aliquot was stopped by the addition of four volumes of cold acetonitrile containing internal standards (100 nM alprazolam, 200 nM imipramine, 200 nM labetalol, and 2 µM ketoprofen). The samples were centrifuged at 3,220 × *g* for 40 min, and an aliquot (90 µL) of the supernatant was mixed with 90 µL of ultrapure H_2_O. The resulting mixture was used for liquid chromatography–tandem mass spectrometry (LC-MS/MS) analysis (Shimadzu and SCIEX Triple Quad 4500). Peak areas were determined from extracted ion chromatograms. A slope, *k*, was determined by linear regression analysis of a plot of the natural logarithm of the remaining percentage of the parent drug versus incubation time. The *in vitro t*_1/2_ (min) was determined from the slope:


$$in\;vitro\;t_{1/2}=-\left(\frac{0.693}{k}\right)$$

*In vitro t*_1/2_ (min, mean of duplicate determinations) was converted to *in vitro* intrinsic clearance (*in vitro* Cl_int_, μL/(min·µg protein)) by means of the following equation :


$$in\;vitro\;Cl_{int}=\left(\frac{0.693}{t_{\frac{1}{2}}}\right)\times \left(\frac{volume\;of\;incubation\;\left(\mu L\right)}{amount\;of\;protein\;\left(\mu g\right)}\right)$$

### *In vivo* metabolism and pharmacokinetics of 6-azasteroid compound 1 and 5

Experiments were performed by Pharmaron, Inc. The metabolism and pharmacokinetics of the 6-azasteroids were studied in CD1 male mice approximately 8 weeks old and 20–30 g. Compounds **1** and **5** were solubilized in 5% DMSO and 5% Solutol in deionized H_2_O with 100 mM citrate buffer (pH 3). Compounds **1** and **5** were administered to mice at a dose of 1 mg/kg by IV or 5 mg/kg PO. Plasma samples were collected at 5, 15, and 30 min and 1, 2, 4, 6, 8, and 24 h after IV administration and at 15 and 30 min and 1, 2, 4, 6, 8, and 24 h after PO administration. Each group contained three mice. Blood samples were collected in anticoagulant tubes containing potassium ethylenediaminetetraacetic acid.

Compound **1** and **5** working solutions (3 µL each of 5, 10, 20, 100, 500, 1,000, 5,000, or 10,000 ng/mL) were separately added to 30 µL of CD1 mouse plasma to achieve calibration standards of 0.5, 1, 2, 10, 50, 100, 500, and 1,000 ng/mL in a total volume of 33 µL. Four quality control samples (1, 2, 50, and 800 ng/mL) for plasma were prepared independently of those used for the calibration curves. These quality control samples were prepared on the day of analysis in the same way as the calibration standards. An aliquot (33 µL) of each standard, quality control sample, and unknown sample (30 µL of plasma combined with 3 µL of blank solution) was added to 200 µL of acetonitrile containing an internal standard mixture for precipitating protein. The samples were vortexed for 30 s and centrifuged at 4°C at 3,220 × *g* for 15 min. The supernatant was diluted three times with water. Diluted supernatant (5 µL) was quantitatively analyzed by LC-MS/MS (API 4000 Triple Quad—AB Sciex, Canada; LC-30A HPLC—Shimadzu, Japan). Non-compartmental pharmacokinetic analysis was performed with Phoenix WinNonlin software (ver. 6.1, Certara, US) using composite data.

### *In vivo* maximum tolerated dose and accumulation studies of 6-azasteroid compound 5

Compound **5** was solubilized in 5% DMSO in 10% Solutol with 100 mM citrate buffer (pH 3) and was tested in Balb/c female mice, approximately 8 weeks old and 20 g, at 100 mg/kg with or without 10 mg/kg rifampicin. Mice (*n* = 3 per group) were treated PO with drug or vehicle once daily at 8 AM. Body weights were measured daily prior to dosing. Mice were observed for physiological signs of toxicity, including unusual or abnormal behavior, poor fur quality, loss of body weight, and death. On day 5, mice were sacrificed at 6 h post-dose, and whole blood and plasma were extracted for complete blood count and blood chemistry profiling on an Element HT5 system (Heska, USA) and plasma drug concentration analysis was performed by LC-MS (Sciex API 4500 Triple Quad, Shimadzu LC-30A). Calibration curves were prepared as described above for *in vivo* metabolism and pharmacokinetics.

### Inhibition of CYP3A4 isoforms

Inhibition of CYP3A4 enzymatic activity was carried out following a standard protocol by Pharmaron, Inc. Human liver microsomes (150 donors pooled, male & female, Corning, Cat# 452117) were used as a source of CYP3A4. The final concentration of human liver microsomes was 0.2 mg/mL. CYP3A4 was tested with testosterone (stock: 10 mM, final: 50 µM) or midazolam (stock: 1 mM, final: 5 µM) as substrate. Aliquots (1 µL) of test compounds (20 µM final concentration), positive control compound ketoconazole (20 µM final concentration), or buffer blank were transferred to a multi-well plate. The reaction was initiated with the addition of 20 µL of 10 mM NADPH solution to provide a final concentration of 1 mM in a 200 µL total volume and carried out at 37°C. The reaction was stopped by the addition of 1.5 volumes of methanol with incubation solution (100 nM alprazolam, 200 nM imipramine, 200 nM labetalol, and 2 µM ketoprofen) at designated time points (5 min midazolam-mediated CYP3A4; 10 min testosterone-mediated CYP3A4). The plate was centrifuged at 3,220 × *g* for 40 min to precipitate protein. Aliquots of 100 µL of the supernatant were diluted with 100 µL ultra-pure H_2_O, and the mixture was used for LC-MS/MS analysis to determine concentration of product (hydroxymidazolam or hydroxytestosterone) formed. The % inhibition was calculated by comparison with no inhibitor control. All experiments were performed in duplicate.

### Inhibition of nuclear receptor binding

Four nuclear receptors: glucocorticoid, androgen, LXRα, and LXRβ were assayed. Inhibition of agonist binding to each of the receptors was carried out following a standard protocol by Eurofins, Inc., USA. Compounds were tested at 10 µM. Compound binding was measured by scintillation counting and was calculated as a % inhibition of the binding of a radioactively labeled ligand specific for each target. Percent inhibition of control specific binding was expressed as


100−[(measured specific binding)/(control specific binding)∗100]

where measured specific binding is the amount of radioactive ligand binding obtained in the presence of test compounds.

The specific cell types and ligands used were (i) GR (human) IM-9 cells (cytosol); agonist radioligand: 1.5 nM [^3^H]dexamethasone (*K*_d_ = 1.5 nM); non-specific negative control: 10 µM triamcinolone; incubation time: 6 h, 4 °C (20). (ii) AR (human) LNCaP cells (cytosol); agonist radioligand: 1 nM [^3^H]methyltrienolone (*K*_d_ = 10.8 nM); non-specific negative control: 1 µM testosterone; incubation time: 24 h, 4 °C (21). (iii) LXRα (human) recombinant protein expressed in *E. coli*; agonist radioligand 20 nM [^3^H] T0901317 (*K*_d_ = 78 nM); non-specific negative control: 10 µM T0901317; incubation time 4 h, 4 °C (22). (iv) LXRβ (human) recombinant protein expressed in *E. coli*; agonist radioligand 20 nM [^3^H] T0901317 (*K*_d_ = 27 nM); non-specific negative control: 10 µM T0901317; incubation time 4 h, 4 °C (23).

### hERG potassium channel inhibition

Inhibition of human ether-a-go-go-related gene (hERG) potassium channel current was carried out following a standard protocol by Pharmaron, Inc. hERG stably expressed HEK 293 cell line (Cat# K1236) was purchased from Invitrogen (Thermo Fisher Scientific, USA). The cells are cultured in 90% DMEM, 10% dialyzed FBS, 0.1 mM NEAA, 25 mM HEPES, 100 U/mL Penicillin-Streptomycin, 5 µg/mL Blasticidin, and 400 µg/mL Geneticin. Cells are split using TrypLE Express about three times a week and maintained between ~40% and ~80% confluence. Before the assay, the cells were plated onto the coverslips at 5 × 10^5^ cells/6 cm cell culture dish and induced with doxycycline at 1 µg/mL for 48 h. Test compound was initially prepared in DMSO with final concentration of 30 mM as stock solution. Then stock solution of test compound was serial-diluted by ratio of 1:3 with DMSO to prepare additional four intermedial solutions including 10, 3.33, 1.11, 0.37 mM, respectively. In order to improve the solubility of test compound, the stock and intermedial solutions were diluted with 4-fold volume of 10% hydroxypropyl beta cyclodextrin (HBCD) and then the working solution was further prepared by dilution of those mixed intermedial solutions containing HBCD with extracellular solution containing 0.1% DMSO in 1000fold so that the final concentration of test compound in working solution was 30, 10, 3.33, 1.11, and 0.37 mM. 5 doses were challenged to the patched cells for IC50 determination.

The voltage-gated hERG potassium channel current was recorded at room temperature (25 °C) from randomly selected transfected cells using whole-cell recording technique with a Multiclamp 700B amplifier (Molecular Devices, US) and an EPC10 microamplifier (HEKA, USA). Extracellular solution (in mM): 132 NaCl, 4 KCl, 3 CaCl2, 0.5 MgCl2, 11.1 glucose, and 10 HEPES (pH adjusted to 7.35 with NaOH). Intracellular solution (in mM): 70 KF, 60 KCl, 15 NaCl, 5 EGTA, and 5 HEPES (pH adjusted to 7.35 with KOH).

The holding potential was set to −90 mV for 1 s; recording current at 50 kHz and filter at 10 kHz. Leaking current was tested at −80 mV for 500 ms. The hERG current was elicited by depolarizing at +30 mV for 4.8 s, and then the voltage was taken back to −50 mV for 5.2 s to remove the inactivation and observe the deactivating tail current. The maximum amount of tail current size was used to determine hERG current amplitude. The current was recorded for 120 s to assess the current stability. Only stable cells with recording parameters above threshold were utilized for the drug administration. First, vehicle control was applied to the cells to establish the baseline. After allowing the current to stabilize for 3 min, compound was applied. hERG current in the presence of test compound were recorded for approximately 5 min to reach steady state and then five sweeps were captured. For dose response testing, compound was applied to the cells accumulatively from low to high concentrations. The positive control Dofetilide was used in this experiment to test the same batch of cells to ensure the good performance of the cells and operations. PatchMaster software (HEKA) was used to extract the peak current from the original data. Percent current inhibition was calculated using the following equation.


$$Peak\;Current\;inhibition=\left[1-{\left(Peak\;tail\;current\right)}_{compound}/{\left(Peak\;tail\;current\right)}_{vehicle}\right]\times 100$$

The dose response curve of test compounds was plotted with % inhibition against the concentration of test compounds using Graphpad Prism 5.0 (Graphpad, USA) and fit the data to a sigmoid dose-response curve with a variable slope. All experiments were performed three times.

### Measurement of *M. leprae* viability and cholestenone production in axenic media

Live *M. leprae* Thai-53 strain was provided by the National Hansen’s Disease Program, Laboratory Research Branch at Louisiana State University in Baton Rouge, LA. Alternatively, the strain was obtained from the hind footpad of nu/nu mice generated at Instituto Lauro de Souza Lima in Bauru, SP, Brazil, and isolated as previously described (24).

*M. leprae* (1 × 10^8^ bacilli) was centrifuged at 12,000 *× g* for 5 min and washed three times with PBS containing 0.05% tyloxapol. Compound **1**, **2,** and **5** were dissolved in 100% DMSO, diluted to the appropriate concentration in 7H9 broth supplemented with 0.1% casein hydrolysate, 0.5% BSA, 0.05% tyloxapol, 50 µg/mL ampicillin, 48 U/mL catalase, and 2% DMSO. Washed *M. leprae* was resuspended with 100 µL of medium or medium containing compound **1**, **2**, or **5**. Bacterial suspensions were incubated at 33°C for 1 h with shaking at 200 rpm. [4-^14^C] cholesterol (final concentration 1 µCi/mL) or/and [1-^14^C] palmitic acid (final concentration 1 µCi/mL) (American Radiolabeled Chemicals, Inc., USA) were added and incubated at 33 °C for 48 h. Radiorespirometry for *M. leprae* viability was performed for samples containing [1-^14^C] palmitic acid (9). Cholestenone production was evaluated by thin layer chromatography (TLC) and phosphor-imaging for samples containing [4-^14^C] cholesterol (9). When [4-^14^C] cholesterol and [1-^14^C] palmitic acid were added simultaneously, the same sample was used for radiorespirometry and TLC of cholestenone. In some experiments, the influence of the compounds on *M. leprae* viability was performed using 1 × 10^7^ bacilli.

### Measurement of NAD^+^ reduction by *M. leprae* lysate

*M. leprae* whole cell lysate (WCL) was obtained by bead-beating gamma-irradiated *M. leprae* Strain NHDP (BEI Resources, NR-19326) with 0.1 mm silica beads and FastPrep bead-beater (MP Biomedicals, USA). Homogenization was carried out in three cycles of agitation at 6.5 m/s for 45 s, with the sample being rested on ice for at least 2 min between cycles. The homogenates were centrifuged at 14,000 *× g* for 5 min to separate the spheres and WCL. The supernatant was recovered, and protein concentration determined using Pierce BCA protein assay (Thermo Fisher Scientific). NAD^+^ (200 µM, MilliporeSigma, USA) reduction was measured as previously described using 50 µg (protein) of WCL protein, with the addition of 200 µM of cholesterol (MilliporeSigma) and in the presence or absence of compound **2** (40 and 100 µM) or compound **5** (100 µM) (10).

### *In vivo* assessment of compound 2 and 5 activity on *M. leprae*

Animal experimentation was conducted under approval by the ILSL ethical committee (CEUA ILSL 02/19). Balb/c female mice with 8-week-old were inoculated with 5 × 10^3^ live *M. leprae* bacilli, strain Thai-53, in both hind footpads, as previously described for the Shepard’s model (25). Eight groups were tested in the experiment: (i) *M. leprae* control group (*n* = 5) inoculated with *M. leprae* and no additional treatment; (ii) vehicle control group (*n* = 4) inoculated with *M. leprae* and treatment with vehicle of the drugs; (iii) regular rifampicin treatment, 10 mg/Kg dose; (iv) sub-inhibitory rifampicin, 1 mg/kg dose (*n* = 5); (v) compound **2** treatment (*n* = 4), 50 mg/kg dose; (vi) compound **2** with sub-inhibitory rifampicin (*n* = 5), 50 mg/kg dose of compound **2** and 1 mg/kg dose of rifampicin; (vii) compound **5** treatment (*n* = 5), 50 mg/kg dose; and (viii) compound **5** with sub-inhibitory rifampicin (*n* = 5), 50 mg/kg dose of compound **5** and 1 mg/kg dose of rifampicin. One month after inoculation, mice from the control group were euthanized and the hind footpad was collected to assess the establishment of the infection by bacillary enumeration. At that time point, vehicle or drug treatment was started for the remaining groups. All compounds were administrated by gavage, rifampicin was administered weekly, and the compounds were administered daily (excluding weekends) for 1 month (20 doses). Mice were euthanized 6 h after administration of the last dose, and blood was collected in heparin-coated tubes by cardiac puncture for blood enzyme evaluation and compound **5** measurement by LC/MS in the plasma. The infected hind footpads from each group of mice were excised, homogenized and bacilli enumerated in a 100 µL aliquot as previously described (26). The remaining homogenate was centrifuged at 12,000 × *g* for 15 min, the supernatant was discarded, and the pellet was suspended in 750 µL of RNA later solution (Thermo Fisher Scientific). The homogenized tissue pellet was incubated at 4°C for 16 h and stored at −80°C until determination of *M. leprae* viability was performed by RT-qPCR (27, 28).

### Assessment of temporal compound 5 concentration in the presence of rifampicin in the plasma of treated mice

Uninfected Balb/c female mice (*n* = 36), approximately 8 weeks old and 20 g, were divided into three groups. One group was kept as the control, and the other two were treated with compound **5** at a dose of 50 mg/kg either alone or in combination with rifampicin at a dose of 1 mg/kg, as described for the *M. leprae* infection studies. At the end of each week, three animals from each group were euthanized, and blood was collected through cardiac puncture in heparin-coated tubes. Concentrations of compound **5** in plasma were determined by LC/MS.

### Liver enzyme detection in the plasma of treated mice

The blood samples obtained from *M. leprae-*infected Balb/c mice were centrifuged at 400 × *g* for 10 min. The resulting plasma was transferred into new 1.5 mL microtubes and kept at −20°C until further analysis. The levels of liver enzymes, aspartate aminotransferase (AST) and alanine aminotransferase (ALT), were subsequently measured in the plasma using colorimetric biochemical assays (Bioclin, Brazil). The assays were carried out following the manufacturer’s instructions.

### Determination of compounds 2 and 5 plasma concentration in Balb/c mice

Compound **5** quantification in the plasma was performed on lipid extracts from plasma samples. Lipid extraction was carried out with a modified Bligh and Dyer method (29) using 50 µL of each plasma sample. The modification in the protocol was included in the wash step by the addition of LC/MS-graded H_2_O, chloroform and 6M HCl in a 1:1:0.07 proportion. The extraction was repeated two additional times, and the organic phases were combined for drying under vacuum with the aid of a SpeedVac (Savant ISS110 Concentrator-Thermo Fisher Scientific).

For LC-MS analysis, samples were reconstituted with 200 µL of chloroform: isopropyl alcohol (1:4) and 20 µL was injected into a Zorbax Eclipse XDB X18 column (Agilent, USA) using an automatic injector, Nexera X2, SIL-30AC (Schimadzu), set at a temperature of 50°C. Compounds **2** and **5** were also injected separately as standards. Liquid chromatography was carried out using a Nexera X2 liquid chromatogram system (Shimadzu) at a flow rate of 0.3 mL/min and mobile phases composed of 0.1% formic acid in water for mobile phase A and isopropyl alcohol for mobile phase B. The gradient was performed with mobile phase A and B at proportions of 90%–10% (0–0.1 min), 20%–80% (10–25 min), and 90%–80% (27–35 min). LC equipment was coupled to a MaXis Impact ESI-Q-TOF mass spectrometer (Bruker, Germany), and samples were analyzed at the positive mode, ionized via atmospheric-pressure chemical ionization (APCI) with 3,000 V of capillary voltage, nebulizing gas pressure at 2 bar, APCI temperature of 350°C, and drying gas temperature of 200°C. The scanning range was from 50 to 800 mass-to-charge ratio (*m*/*z*). Data analysis was carried out on the QuantAnalysis software version 2.2 (Bruker).

### *M. leprae* molecular viability

For assessment of *M. leprae* viability, the homogenized tissue pellets were transferred to tubes containing 0.1 mm silica spheres, and 1 mL of TRIzol reagent (Thermo Fisher Scientific) and mycobacterial cell wall was further disrupted with the aid of the FastPrep equipment (MP biomedical) at 6.5 m/s for 45 s. RNA and DNA extractions were performed as previously described (10, 27). RNA samples were treated with the Turbo DNA-free kit (Thermo Fisher Scientific), and 1,000 ng of DNAse-treated RNA was then used to synthesize the cDNA using the GoScript kit (Promega, USA). *M. leprae* molecular viability was determined by RT-qPCR, as previously described (27, 28), using 10 ng and 20 ng of cDNA and DNA samples, respectively, and the *M. leprae* rRNA 16S gene as the molecular target.

### Statistical analysis

All statistical analyses were performed using GraphPad Prism 8.0 (GraphPad, USA). Normality of the data sets was assessed using the Shapiro-Wilk test. For data sets that followed a normal distribution, parametric tests were applied. Multiple group comparisons were conducted using one-way ANOVA with Tukey’s post hoc test for comparisons across all groups, or Bonferroni’s post hoc test when comparisons were made against a specific reference group. For data sets that did not follow a normal distribution, non-parametric tests were used: the Kruskal-Wallis test for non-paired data sets and the Friedman test for paired data sets, both followed by Dunn’s multiple comparison test as the post hoc analysis. Statistical significance was defined as a *P-*value less than 0.05.

## RESULTS AND DISCUSSION

Six 6-azasteroid compounds with modifications to the steroidal nucleus and side chain were synthesized in our laboratory (Fig. 1; Table 1) followed by characterization of their pharmacologic properties.

**TABLE 1:** Overview of 6-azasteroid modifications, stability, and cytotoxicity

	Carbamoyl substituent	6 N substituent	C_4_-C_5_	cLogP	HLM stability *t*_1/2_ (min)^*a*^	Mammalian cell IC50 (μM) Cytotoxicity^*b*^(18)	
						THP1	HepG2
1	(2, 5-di-*t*-butyl)anilide	H	alkene	6.2	45	18	ND^*c*^
2	(2, 5-di-*t*-butyl)anilide	propyl	alkene	7.4	18	24	21
3	(2,5-di-trifluoromethyl)anilide	4-fluoropropane	alkyl	6.4	45	ND	>100
4	(2,5-di-trifluoromethyl)anilide	3-trifluoromethyl ethane	alkyl	7.6	63	ND	>100
5	(4,4′-difluoro)diphenylmethyl[carbamoyl]	H	alkene	4.9	443	24	31
6	(4,4′-difluoro)diphenylmethyl[carbamoyl]	propyl	alkene	6.1	337	ND	>100

^*a*^ 6-azasteroid (1 μM) was incubated with human liver microsomes (HLM) (0.5 mg/mL, taken from a mixed gender pool) at 37°C for 1 h. Reported half-lives are the mean of duplicate measurements.

^*b*^ Cytotoxicity as determined in the indicated cell type (18).

^*c*^ ND, not determined.

### Stability of 6-azasteroids in human liver microsomes and cytotoxicity

We initially assessed the metabolic stability of the six 6-azasteroid compounds using pooled human liver microsomes. The 6-azasteroid compounds **1** and **2** with a (2,5-di-*tert*-butyl) anilide substituent on the side chain had poor microsomal stability because the di-*tert*-butyl groups were highly metabolically vulnerable to terminal hydroxylation (Table 1). This result is consistent with literature relating to the metabolism of a structurally similar 4-azasteroid, finasteride, which has a *tert*-butyl R_1_ group (30). In addition, substitution of the 6-nitrogen with an alkyl group (compound **2** and **6**) resulted in a shorter half-life, presumably due to the susceptibility of the 6-substituent to metabolism (Table 1).

Replacement of di-*tert*-butyl groups with one or more metabolically stable trifluoromethyl groups in combination with reduction of the C4-C5 alkene, and substitution of the 6-nitrogen with fluorinated alkyl chains (compounds **3** and **4**) only slightly improved *in vitro* stability. Our observations are consistent with the metabolism data for the commercial 4-azasteroid dutasteride, which has a 2,5-ditrifluoromethyl-substituted anilide moiety (31). Preclinical and clinical data have shown that this 4-azasteroid undergoes anilide 4′-hydroxylation as well as C/D-ring hydroxylation, both processes are mediated by CYP450 enzymes (31).

To overcome these metabolic limitations, we selected a (diphenylmethyl) carbamoyl system for the R_1_ side chain with 4-fluoro phenyl substituents to avoid potential metabolic vulnerability of the aromatic ring. The introduction of the (diphenylmethyl)carbamoyl moiety positively impacted microsomal stability. Compounds **5** and **6** displayed markedly longer half-life (*t*_1/2_) values nearly 10 times the *t*_1/2_ of the 2,5-di-*tert*-butyl analog (compounds **1** and **2**). The addition of the alkyl group at the 6-nitrogen (compound **6**) did not have a major impact on metabolic stability, which indicates that this carbamoyl side-chain blocked cytochrome P450 activity.

Previously, we demonstrated that azasteroids with an unsaturated A/B-ring system generally displayed cytotoxicities between 100 and 10 µM when they had either a 2,5-disubstituted anilide or a (diphenylmethyl)carbamoyl side chain (18). Saturation of the vinylogous amide spanning the A/B-ring system concentrates electron density at the 6-position nitrogen, increasing its nucleophilicity and thereby increasing its affinity for off-target enzymes. Compounds with a protonated 6-position nitrogen generally displayed the highest cytotoxicities compared to compounds in which the 6-position nitrogen was alkylated. Consistent with our prior observations (17), compounds **3** and **4** with a 2,5-di-trifluoromethyl)anilide side chain and alkylated at the 6-position nitrogen exhibited very low cytotoxicities. The cytotoxicities we observed for compounds **5** and **6** with a (diphenylmethyl)carbamoyl side chain followed this trend although the alkene was unsaturated (Table 1). However, introduction of a propyl group at the 6-position nitrogen increased the cLogP by an order of magnitude. Upon consideration of all the metabolic properties measured, we selected compound **5** as our lead molecule due to its optimal combination of long microsomal half-life, mid-micromolar cytotoxicity, and moderate cLogP.

### Pharmacokinetics of 6-azasteroids in mice

We elucidated the *in vivo* pharmacokinetics of the lead molecule (compound **5**) that had a half-life in human liver microsomes greater than 6 h, as well as that of the initial compound **1** in order to provide a baseline comparison of mouse *in vivo* pharmacokinetic behavior, given its structural similarities with other compounds. It is worth mentioning that only two compounds were evaluated in this experiment to minimize animal testing. Healthy CD1 male mice were administered 6-azasteroid compound **1** or **5** by IV (1 mg/kg) or oral gavage (PO, 5 mg/kg) and then euthanized at specified time points over 24 h. Plasma was extracted for analysis of drug concentrations.

The *in vivo t*_1/2_ values for the two compounds mirrored their microsomal stabilities (Table 1); the *t*_1/2_ for compound **5** was approximately 2.5-fold longer than for compound **1** (Table 2; Fig. 1B). The *C*_max_ value of compound **5** was favorable and four times greater than for compound **1**. Compound **1** underwent rapid clearance from plasma after PO administration; most of the compound was cleared from the plasma by 4 h. Clearance of compound **5** was slower from plasma: more than 50% of the compound remained after 6 h, and the plasma concentration slowly decreased between 6 and 24 h.

**TABLE 2:** *In vivo* pharmacokinetic parameters of 6-azasteroids **1** and **5**^*a*^

		Compound	
		1	5
5 mg/kg PO			
*t*_1/2_	h	1.0	2.8
*T*_max_	h	0.5	0.5
*C*_max_	ng/mL	501	2,210
AUC_inf_	h·ng/g	822.1	18,566
*F*	%	46.2	118.9
1 mg/kg IV			
Cl_obs_	(mL/min)/kg	46.8	5.34
*T*_max_	h	0.642	2.60
*C*_max_	ng/mL	803	838
AUC_inf_	h·ng/g	356	3,123

^*a*^ CD1 male mice (8 weeks, 20–30 g) were dosed as indicated. Abbreviations and symbols: PO, oral gavage; *t*_1/2_, half-life; *T*_max_, time to reach *C*_max_; *C*_max_, maximum drug concentration; AUC, area under curve; *F*, bioavailability; Cl_obs_, observed clearance.

Compound **5** displayed a favorable bioavailability profile: 119% was found in circulation in the blood upon PO administration when compared to IV-administered drug-plasma concentrations. This profile was substantially better than that of compound **1** (46.2% plasma bioavailability). It is important to note that the IV doses of compounds **1** and **5** were one-fifth the PO doses; PO and IV doses likely do not scale in direct proportion, which could lead to variable results. The high bioavailability of compound **5** and the short PO *T*_max_ values of compounds **1** and **5** are likely due to the low calculated partition coefficients (cLogP, between octanol and water) of 6.2 and 4.9, respectively (Table 1).

Tolerance of maximally solubilized doses at 100 mg/kg of compound **5** was determined with a 5-day dosing regimen. Dosing was performed with and without administration of a therapeutic 10 mg/kg dose of co-drug rifampicin (Fig. S1A). This was done based on the knowledge that rifampicin can induce both gut (enterocytic) and liver (hepatic) CYP450 activity, especially CYP3A4 (32), which is a key enzyme responsible for metabolism of azasteroids (31). Therefore, rifampicin can increase its own metabolism (autoinduction) and may, therefore, increase the metabolism of azasteroids, reducing efficacy. We found that azasteroid compound **5** concentration was not affected by co-administration of rifampicin. This may be because compound **5** is itself an effective inhibitor of CYP3A4 activity as shown in Table 3. Six hours after the 5th daily dose, the plasma concentration of compound **5** was 292 ± 59 µM when administered alone and 274 ± 80 µM when co-administered with rifampicin. This small difference was not statistically significant (Fig. S1B). In addition, blood chemistry parameters (Fig. S1C) and the complete blood count (Fig. S2) were measured after the 5th daily dose, and no significant differences were observed between compound **5** alone and co-administration with rifampicin.

**TABLE 3:** *In vitro* pharmacology of 6-azasteroids

		% inhibition observed		
Target tested	Concentration tested	1	2	5
Cyp3A4 Midazolam Isoform	20 µM	ND^*a*^	ND	79%
Cyp3A4 Testosterone Isoform	20 µM	ND	ND	68%
Glucocorticoid Receptor	10 µM	ND	ND	46.4
Androgen Receptor	10 µM	ND	ND	9.1
LXRα	10 µM	53	64	11
LXRβ	10 µM	72	69	26
		IC_50_		
hERG^*b*^ channel		0.95 µM	0.74 µM	3 µM

^*a*^ ND, not determined.

^*b*^ hERG, human ether-a-go-go-related gene potassium channel.

An extended experiment was performed with non-infected mice to monitor plasma levels of compound **5** either alone or in combination with rifampicin at the sub-dose of 1 mg/kg over 4 weeks of treatment. Plasma measurements were obtained at weekly time points, and no significant differences were observed between treated groups (Fig. S3).

### Additional pharmacological parameters for azasteroids

We tested whether compound **5** binds to four different nuclear receptors, CYP3A4 and the hERG potassium channel, all of which are common targets of hydrophobic or steroidal drugs like 6-azasteroids (Table 3). We found that azasteroid **5** strongly inhibits the enzymatic activity of both isoforms of CYP3A4 consistent with prior reports that azasteroids are substrates of CYP3A4 (31). In contrast, compound **5** shows low binding to the androgen, LXRα, and LXRβ nuclear receptors. Binding of this compound to glucocorticoid receptor was moderate. In contrast, compounds **1** and **2** significantly inhibit binding of the synthetic ligand T0901317 to LXRα and LXRβ receptors, and testing of additional receptors was not pursued. Azasteroids **1** and **2** showed higher inhibition activity over hERG when compared to compound **5**, highlighting how the (2, 5-di-*t*-butyl)anilide side-chain is a poor pharmacophore due to multiple liabilities. Introduction of the diphenylmethylcarbamoyl side-chain reduced hERG liabilities and reduced nuclear receptor binding. Although compound **5** is a nanomolar inhibitor of human type 1 and type 2 5α-reductase (13), further adjustment of substituents on the steroid scaffold may enable optimization of pharmacologic properties. For example, substitution of N-6 with a propyl group or Δ^1^ unsaturation or 1,2-cyclopropanation have been shown to diminish potency against 5α-reductase (13). Alternatively, topical formulations that minimize systemic liabilities may be pursued to improve pharmacologic properties with potential application particularly in paucibacillary leprosy (33).

### Effects of 6-azasteroids 2 and 5 on *M. leprae* 3β-HSD activity

**Fig 2:**
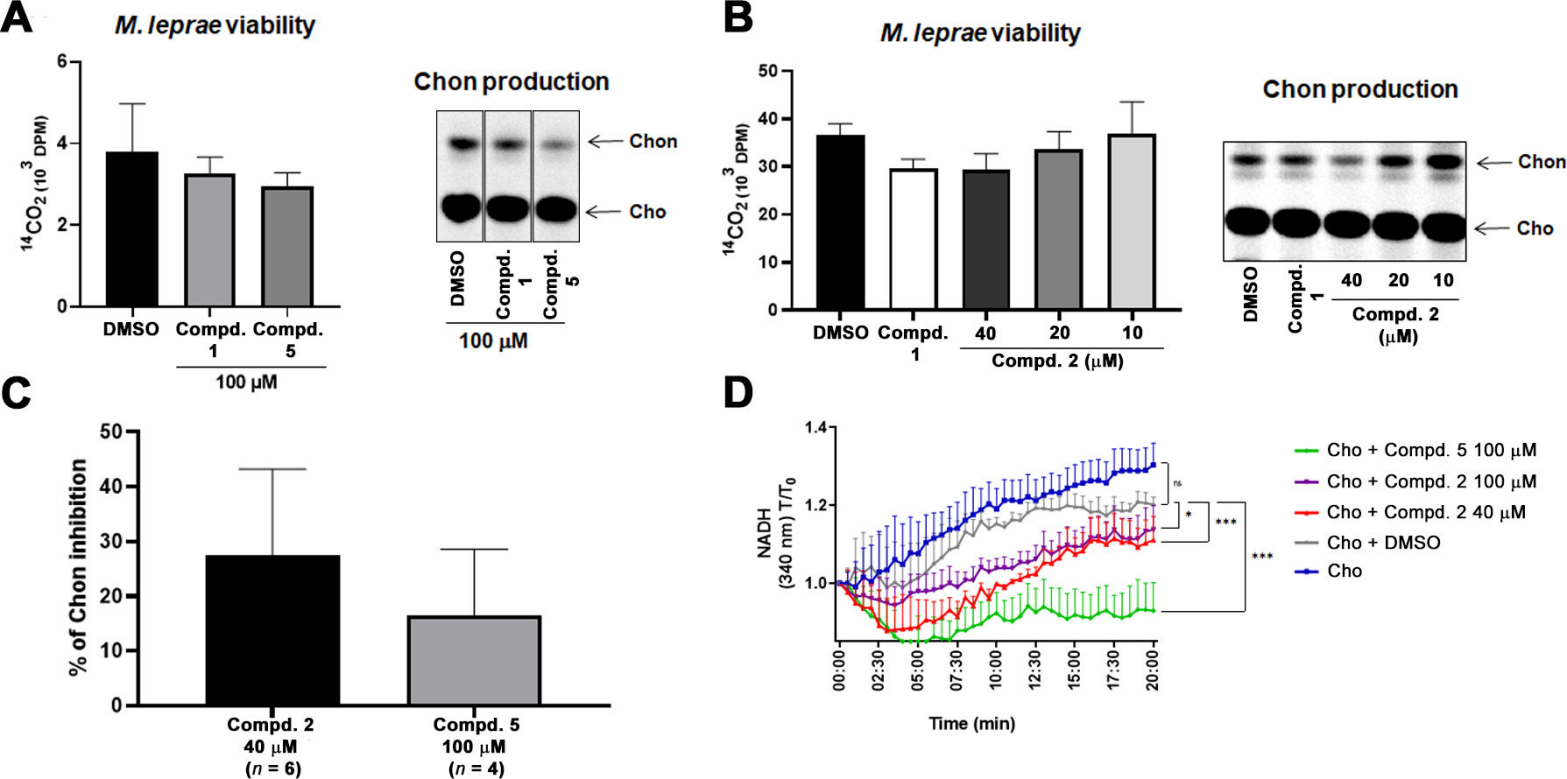
Effect of the 6-azasteroids on *M. leprae* 3β-HSD activity. *M. leprae* was pre-treated with compound **5** (**A**) or compound **2** (**B**) for 1 h at 33°C followed by incubation for 48 h with [1–^14^C]palmitic acid and/or [4–^14^C] cholesterol. *M. leprae* viability was estimated by radiorespirometry, and cholestenone production was evaluated by thin layer chromatography (TLC). 3β-HSD inhibition (**C**) was estimated by densitometric TLC analysis of 4–6 biological replicates. *M. leprae* lipid extracts were resolved by TLC with solvent chloroform-methanol (95:5). TLC plates were exposed to the phosphor screen and radiolabeled compounds were visualized by PhosphorImager analysis. Standards were visualized by spraying the TLC plates with anisaldehyde solution and heating. Densitometry analysis of the chromatogram was performed using the Image QuantTL program. (**D**) *M. leprae* WCL was incubated with cholesterol alone (Blue) or with cholesterol together with treatment with 6-azasteroids (Compd. **2** 40 µM—Red; Compd. **2** 100 µM—Purple, and Compd. **5** 100 µM—Green) or its vehicle DMSO (gray), and NADH generation was measured at 340 nm 30 s for 20 min. Each point represents mean values of the ratio absorbance *T*/ absorbance *T*_0_ with SEM (*n* = 4). Statistical analysis was performed by applying the non-parametric and paired test of Friedman with dunn’s multiple comparisons test using the cholesterol +DMSO condition as the reference for each group comparison, ns (not significant); *(*P* < 0.05); **(*P* < 0.01); and ***(*P* < 0.001). Compd, compound; Cho, cholesterol; Chon, cholestenone.

We previously showed that compound **1** inhibits *M. leprae* 3β-HSD activity as measured by the reduction of cholestenone production by *M. leprae* during cultivation in axenic medium (10). In the present study, we extended this analysis to compound **5** based on its promising pharmacological parameters and included compounds **1** and **2** for comparison. It is important to note that the minimal structural changes observed in compound **2** compared to compound **1** (Fig. 1; Table 1) present an interesting opportunity to broaden our analysis by exploring the effects of these compounds on *M. leprae* 3β-HSD activity. *M. leprae* was incubated for 24 h with the compounds, and bacterial viability and cholestenone production were determined by radiorespirometry and TLC, respectively. Depending on the amount of *M. leprae* available, cholesterol and palmitic acid were added together in the same tube, or in separate tubes, without interfering with the results obtained. Initially, all compounds were tested using the 6-azasteroid concentrations of 100 µM, the optimal concentration previously determined for compound **1** (10). At this concentration, compound **5** inhibited 3β-HSD activity without significantly affecting bacterial viability (Fig. 2A). However, compound **2** at this concentration drastically reduced *M. leprae* viability as well as consequently cholestenone production (Fig. S4). Lower concentrations of compound **2** were tested, and the concentration of 40 µM was adopted for further *in vitro* experiments as it did not significantly affect *M. leprae* viability (Fig. 2B). Compound **2** (40 μM) and compound **5** (100 μM) inhibited cholestenone production by *M. leprae* by approximately 38% (σ16.33) and 25.05% (σ7.95), respectively (Fig. 2C). The high variability observed for this assay was attributed to the complexity of this assay involving multiple steps, radioactive material, and most notably differences in bacterial viability among the different batches of nude mouse-derived *M. leprae*. It is also worth emphasizing that the 6-azasteroid concentrations chosen in these assays were those not reducing bacterial viability, thus the decrease in cholestenone production was only attributed to the inhibition of 3β-HSD, not to a decrease in the number of viable bacteria.

To further investigate the effects of compounds **2** and **5** on *M. leprae* 3β-HSD activity, WCL was treated with each compound and the NAD^+^ reduction in the presence of cholesterol was measured. Similar to what we reported for compound **1** (10), both compound **2** (40 µM: mean value = 0.9986, *P* < 0.0001 and 100 µM: mean value = 1.038 , *P* = 0.0107) and **5** (100 µM: mean value = 0.9070, *P* < 0.0001) decreased the rate of NAD^+^ reduction in comparison to the condition treated only with the vehicle (DMSO, mean value = 1.119), confirming their efficiency in inhibiting 3β-HSD enzyme activity (Fig. 2D). It is important to note that compound **5** inhibited NADH generation more remarkably than compound **2**. Overall, these results support the conclusion that both compound **2** and **5** inhibit *M. leprae* 3β-HSD activity.

### 6-Azasteroid impact on *M. leprae* survival in the Shepard’s model

**Fig 3:**
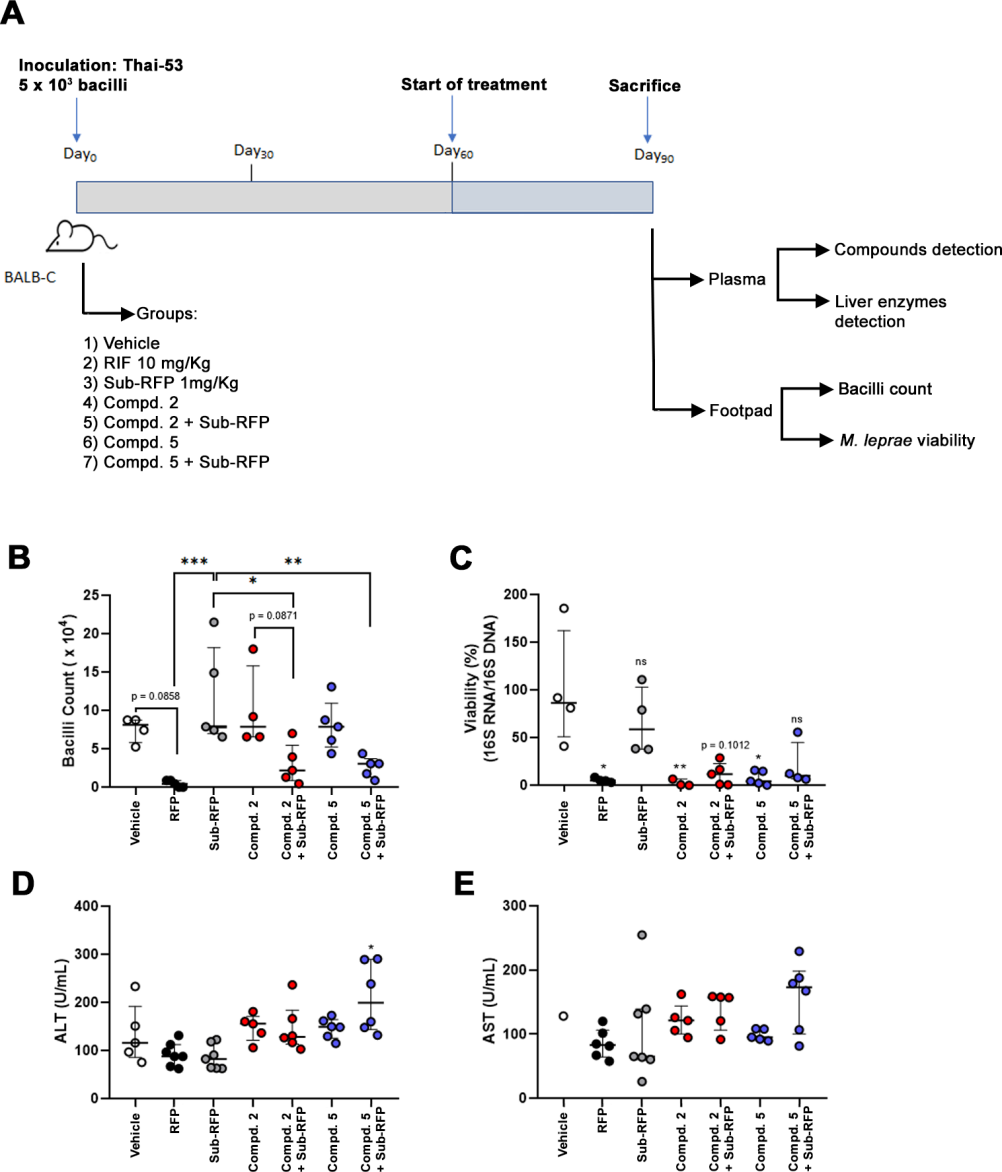
6-Azasteroids impact *M. leprae* growth and viability *in vivo*. (**A**) Schematic representation of the experiment design. Balb/c female mice (8 weeks, 20 g) were inoculated with 5 × 10^3^ live *M. leprae* Thai-53 strain in both hind footpads at day 0. At day 60, the treatment with the 6-azasteroids (Compd. **2**, *n* = 4; Compd. **2** + sub-RFP, *n* = 5; Compd. **5**, *n* = 5, and Compd. **5** + RFP sub, *n* = 5), rifampicin (RFP, *n* = 5 and sub-RFP, *n* = 5), or drug vehicle (vehicle, *n* = 4) was initiated via oral gavage, until day 90, when mice were euthanized for determination of bacilli count (**B**) and *M. leprae* molecular viability (**C**) in footpad homogenates, and determination of liver enzyme levels, ALT (**D**) and AST (**E**), in the plasma. Compd: compound. (**B**) Bacilli count was determined by microscopy via analysis of 60 fields in acid-fast stained slides. Statistical analysis was performed using a one-way ANOVA, followed by Tukey’s multiple comparisons test across all groups, *(*P* < 0.05), **(*P* < 0.01), and ***(*P* < 0.001). (**C**) *M. leprae* molecular viability was determined by analyzing the ratio of cDNA/DNA for ML 16S rRNA gene by RT-qPCR. Statistical analysis was performed for each group in comparison to the vehicle group as the reference using the non-parametric Kruskal-Wallis test, followed by Dunn’s test for correction for multiple comparisons, ns, not significant, *(*P* < 0.05) and **(*P* < 0.01). (**D, E**) Liver enzymes were determined in the plasma by colorimetric biochemical assay and statistical analysis was performed using a one-way ANOVA, with comparisons made between each group and the vehicle group as the reference, followed by Bonferroni’s multiple comparisons test (*P* < 0.05).

In order to study the *in vivo* effects of 6-azasteroids on *M. leprae,* we adopted Shepard’s mouse footpad model for bacterial growth in the Balb/c mouse (25). Compounds **2**, **5** or rifampicin (included as a positive control) were administered to the Balb/c female mice starting 2 months after infection of the hind footpads with 5 × 10^3^ live *M. leprae* and continued for 30 days, after which the animals were euthanized (Fig. 3A). It is worth mentioning that compound **2** was included in the *in vivo* assay as a reference for comparison with compound **5**. This decision was made due to its structural similarity to compound **1**, which has already been demonstrated to reduce the intracellular viability of *M. leprae in vitro* (10). Additionally, the results of this current study regarding compound **2**’s performance in axenic medium assays were also considered. In some groups of animals, treatment was carried out with a sub-inhibitory dose of rifampicin (1 mg/kg) in combination or not with the azasteroids to check potential interaction between these drugs. After 1 month of treatment, the standard dose of rifampicin (10 mg/kg) reduced the bacillary load (median = 0.438 × 10^4^, *P* = 0.0858) compared to vehicle-treated animals (median = 8.110 × 10^4^). The rifampicin sub-inhibitory dose did not significantly reduce the bacillary load (median = 7.890 × 10^4^, *P* = 0.6280) in comparison to the group treated with the vehicle (Fig. 3B). The treatment of *M. leprae* infected mice with compound **2** or **5** alone did not affect bacterial numbers when compared to the vehicle control (compound **2**: median = 7.885 × 10^4^; compound **5**: median = 7.890 × 10^4^). In contrast, when these azasteroids were co-administered with the sub-inhibitory dose of rifampicin reduction in bacillary load was observed, and it presented statistical significance when compared to the group treated with the sub-optimal dose of rifampicin (compound **2** + sub-RFP =2.190 × 10^4^, *P* = 0.0122; compound **5** + sub-RFP = 3.070 × 10^4^, *P* = 0.0084) (Fig. 3B). Reinforcing these data, at the end of the experiment with infected mice, detectable levels of compounds **2** and **5** were still present in the plasma with no remarkable differences between the groups treated with compound alone or in combination with rifampicin (Fig. S4B and C).

To complement the enumeration of bacilli by microscopy, molecular viability analyses were performed. This revealed that treatment with either compound **2** or **5** alone significantly reduced *M. leprae* viability as compared to the vehicle control (compound **2**: median viability = 0.5442%, *P* = 0.0078; compound **5**: median viability = 4.205%, *P* = 0.0297). As expected, a reduction in bacterial viability was also observed in mice treated with the sub-inhibitory dose of rifampicin in combination with compound **2** or **5** (Fig. 3C). The drug treatments had no apparent hepatotoxic effect, as there were no differences in the plasma levels of the enzymes ALT (Fig. 3D) or AST (Fig. 3E).

The notable difference in drug performance when bacilli count vs molecular viability was measured deserves an explanation. In contrast to the molecular viability assay, bacilli counting under light microscope cannot precisely differentiate metabolically active from dead bacteria and may underestimate the potential killing effect of a therapeutic regimen. The short-term treatment of 1 month adopted in our assays also probably contributed to the different results observed between the two methods by giving insufficient time for bacterial clearance. The bacilli count suggests that the effect of 6-azasteroids on *M. leprae* is more gradual than rifampicin, which has a strong and rapid effect (34), resulting in a delay in bacterial clearance. However, the molecular viability revealed that by the end of the 1-month treatment period, the azasteroid effect seemed to reach its peak, as all bacteria were metabolically inactive and not viable whether treated with 6-azasteroids alone or in combination with rifampicin. Therefore, it is possible that bacteria counting underestimates the effect of 6-azasteroid alone on *M. leprae* survival. However, bacteria count allowed us to note that the combination of the azasteroid compound with a sub-dose of rifampicin clearly enhances the effect of rifampicin, leading to a more rapid bacterial killing.

Moreover, in the current study, although there were no significant differences in the *in vivo* and *in vitro* data between the two compounds tested, the combination with pharmacokinetics data indicates that compound **5** is a more promising candidate for future studies. Taken together, the results from the current *in vivo* experiment expand previous observations from our group that demonstrated the crucial role of 3β-HSD for *M. leprae* viability and the establishment of the infection. Although inhibition of *M. leprae* 3β-HSD may not be the only activity responsible for the killing effects of compound **5** *in vivo* and inhibition of host targets may be contributing to the killing effect, this may add more value to the compound. Targeting host proteins may avoid the emergence of resistant strains (35) or may counteract the effects of bacterial metabolites on the host immune system (36). It is important to note that azasteroids may also affect steroid hormone pathways and could present age- and sex-specific risks (37) that were not examined in our study. Similar drugs, such as finasteride and dutasteride, have been successfully used to treat various androgen-related conditions (38). Despite inhibiting 5α-reductase (13) and the associated side effects, these drugs may offer valuable adjunctive effects in managing leprosy, provided there is careful monitoring. This also highlights a crucial target for drug development using 6-azasteroid compounds in the case of *M. leprae* infection, which requires further investigation.

### Conclusion

We established structure–activity relationships with respect to novel side chains and some small modifications to the azasteroid scaffold, which improved the stability of compounds in human liver microsomes and translated to improved accumulation and extended *t*_1/2_ values in mice in comparison to the initially tested lead 6-azasteroid analog. Azasteroid **5** has low metabolic liabilities in comparison to its progenitors azasteroids **1** and **2**. Azasteroid **5** was also able to inhibit *M. leprae* 3β-HSD *in vitro* and showed an effective killing capacity *in vivo* both alone or in synergism with rifampicin without detectable hepatotoxic effects. We conclude that 6-azasteroids derivatives emerge as promising new antimicrobial candidates for leprosy treatment; however, further structural modifications are needed to minimize inhibition of mammalian targets and maximize anti-leprosy activity.

## SUPPLEMENTAL MATERIAL

Supplemental material spectrum.00228-25-s0001.pdf Fig. S1 to S4.

ASM does not own the copyrights to Supplemental Material that may be linked to, or accessed through, an article. The authors have granted ASM a non-exclusive, world-wide license to publish the Supplemental Material files. Please contact the corresponding author directly for reuse.
