# Supplementary material for: Pharmacologic properties and inhibitory activity of 6-azasteroids against Mycobacterium leprae in vivo and in vitro
Source: Microbiol Spectr. 2025 May 27;13(7):e00228-25. doi: 10.1128/spectrum.00228-25 (PMC12210912; doi:10.1128/spectrum.00228-25)
Supplement: Supplemental material — Fig. S1 to S4. [file spectrum.00228-25-s0001.pdf]

Supplementary figures

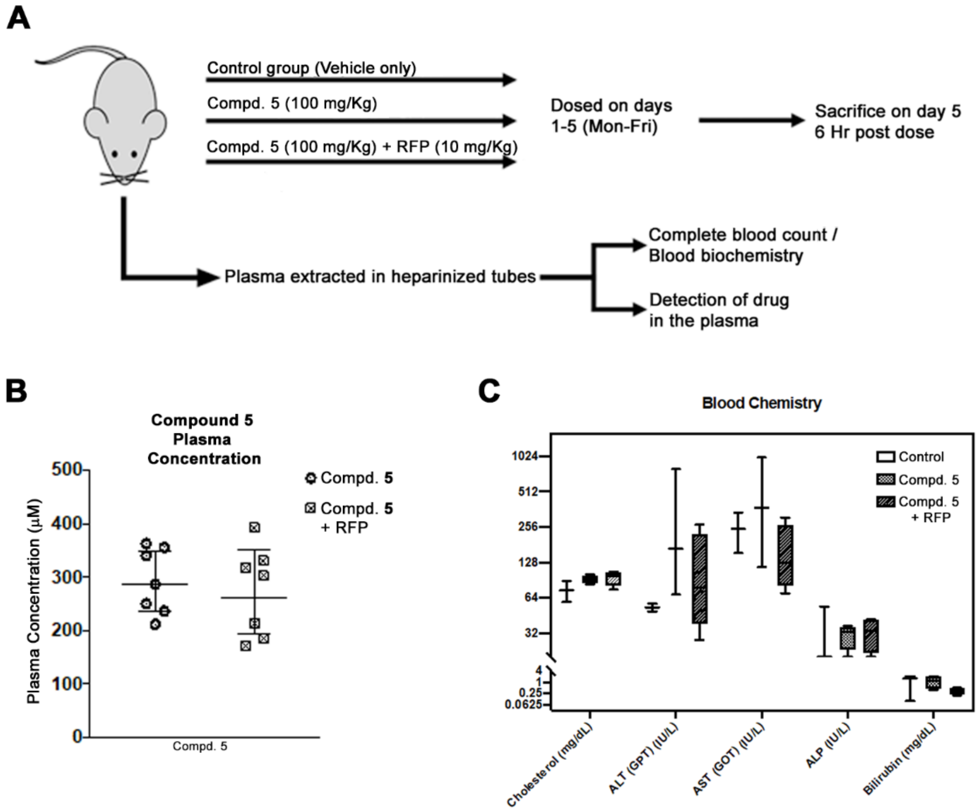

Supplementary Figure 1. Maximum tolerated dose (MTD) experiment to assess compound 5 toxicity and plasma concentration. (A) Experiment design for MDT assay, *n*=7 Balb/c female mice (8 weeks, 20 g) for each group. (B) Compound **5** detection in the plasma by LC-MS. (C) Biochemical assay for MTD experiment of compound 5**.** Cholesterol, bilirubin, and enzymes (ALT, AST and ALP) were detected in the plasma. Compd: compound; RFP: Rifampicin.

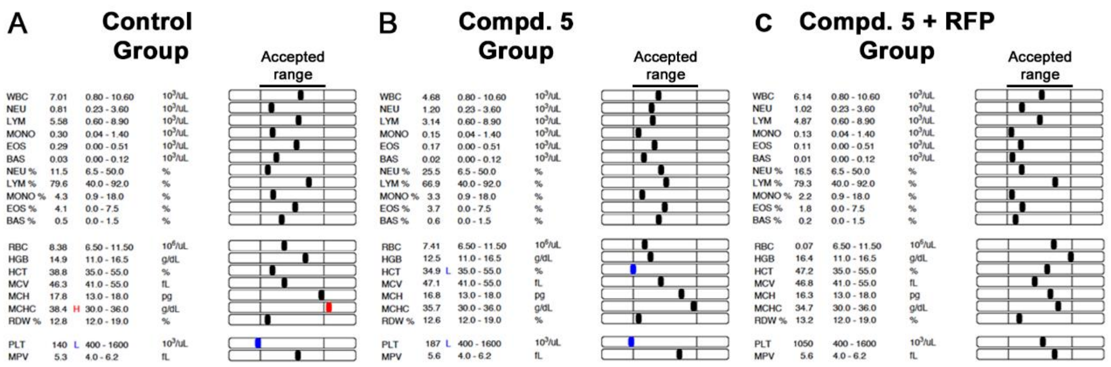

Supplementary Figure 2. Complete blood count from mice from MTD experiment of compound 5. WBC: White blood cells; NEU: Neutrophil; LYM: Lymphocyte; MONO: Monocyte; EOS: Eosinophil; BAS: Basophil; NEU%: % of neutrophils; LYM%: % of lymphocytes; MONO%: % of monocytes; EOS%: % of eosinophil; BAS%: % of basophil; RBC: Red blood cells; HGB: Hemoglobin; HCT: Hematocrit; MCV: Mean corpuscular volume; MCH: Mean corpuscular hemoglobin; MCHC: Mean corpuscular hemoglobin concentration; RDW%: % of red cell distribution width; PLT: platelets; MPV: mean platelet volume. Compd: compound; RFP: Rifampicin. Values below the accepted range are indicated in blue (L) and values above are indicated in red (H).

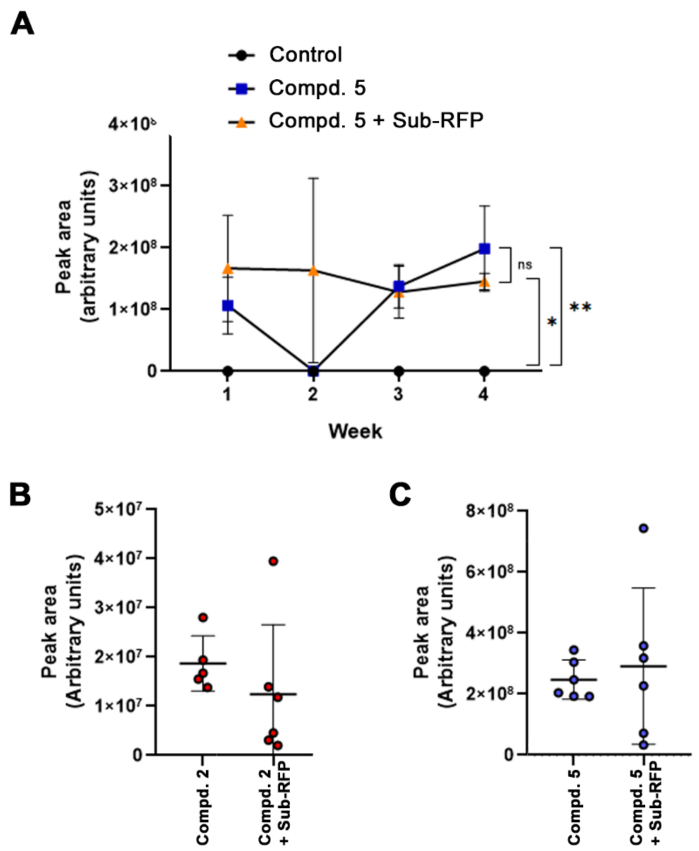

Supplementary Figure 3. 6- Azasteroids quantification in the plasma of Balb/c female mice. (A) Compound detection analysis was performed for compound **5** alone (50 mg/kg compound **5**, blue) or in combination with rifampicin at a dose of 1 mg/kg (compound **5** + sub-RFP, orange), comparing with no treatment (Control, black), during 4 weeks of oral gavage given daily Mon–Fri. Animals were euthanized by the end of each week, 6 hours after the last drug dose and blood samples were collected by cardiac puncture. Lipids were extracted from the plasma and compound **5** was detected in the plasma by LC-MS. Statistical analysis was performed by applying ANOVA with Turkey's multiple comparisons test across all groups, ns (non-significant); *($p<0.05$) and **($p<0.01$). (B, C) Blood samples collected from *M. leprae*-inoculated Balb/c were centrifuged to obtain plasma. Lipids were extracted from the plasma samples and

compound **2** (B) and compound **5** (C) were detected in the plasma by LC-MS. Compd: compound.

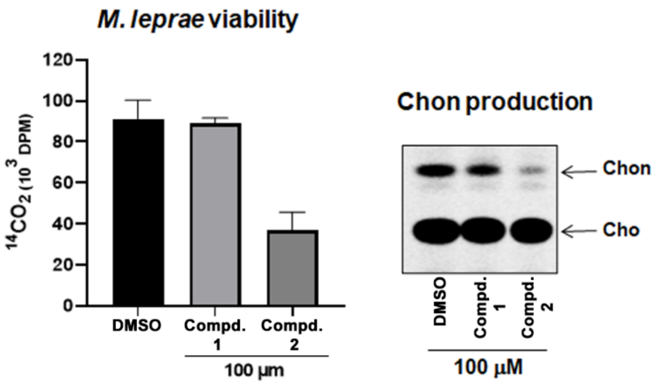

Supplementary Figure 4. Effect of the 6-azasteroids on cholestenone production by *M. leprae* in axenic medium. *M. leprae* was pre-treated with 100 µM compound **2** for 1 h at 33 °C followed by incubation for 48 h with [1-^{14}C] palmitic acid and/or [4-^{14}C] cholesterol. *M. leprae* viability was estimated by radiorespirometry and cholestenone production was evaluated by thin layer chromatography (TLC); Compd: compound; Cho: cholesterol; Chon: cholestenone.
